# The Emerging Role of BDNF/TrkB Signaling in Cardiovascular Diseases

**DOI:** 10.3390/life11010070

**Published:** 2021-01-19

**Authors:** Peng-Zhou Hang, Hua Zhu, Pei-Feng Li, Jie Liu, Feng-Qin Ge, Jing Zhao, Zhi-Min Du

**Affiliations:** 1 Department of Pharmacy, Clinical Medical College, Yangzhou University, Northern Jiangsu People’s Hospital, Yangzhou 225001, China; 18051063961@yzu.edu.cn (P.-Z.H.); 18051061331@yzu.edu.cn (H.Z.); MZ120201712@yzu.edu.cn (F.-Q.G.); 2 Institute of Clinical Pharmacology, The Second Affiliated Hospital of Harbin Medical University (University Key Laboratory of Drug Research), Harbin 150086, China; lipeifeng0224@163.com (P.-F.L.); lj20201010@163.com (J.L.); 3 Medical Research Center, Clinical Medical College, Yangzhou University, Northern Jiangsu People’s Hospital, Yangzhou 225001, China; 4 State Key Laboratory of Quality Research in Chinese Medicines, Macau University of Science and Technology, Macau 999078, China

**Keywords:** brain-derived neurotrophic factor, tropomyosin-related kinase receptor B, cardiovascular diseases, biomarker

## Abstract

Brain-derived neurotrophic factor (BDNF) is one of the most abundantneurotrophins in the central nervous system. Numerous studies suggestthat BDNF has extensive roles by binding to its specific receptor, tropomyosin-related kinase receptor B (TrkB), and thereby triggering downstream signaling pathways. Recently, growing evidence highlightsthat the BDNF/TrkB pathway is expressed in the cardiovascular system andclosely associated with the development and outcome of cardiovascular diseases (CVD), including coronary artery disease, heart failure, cardiomyopathy, hypertension, and metabolic diseases. Furthermore, circulating BDNF has also been revealed as a new potential biomarker for both diagnosis and prognosis of CVD. In this review, we discuss the current evidence of the emerging role of BDNF/TrkBsignalingand address the challenges that remain in translating these discoveries to novel therapeutic strategies for CVD.

## 1. Introduction

The neurotrophin (NT) family is generally reserved for four structurally related factors: neuronal growth factor (NGF), brain-derived neurotrophic factor (BDNF), NT-3, and NT-4/5 [1]. Among them, BDNF is a protein isolated and firstly purified from pig brain in 1982 [2]. Subsequently, the expression of BDNFwasalso detected in the cardiovascular system [3]. Nowadays, it has been well recognized that BDNF is the most abundant endogenous neurotrophic factor in the body [4]. Studies have shown that BDNF is a master regulator ofsynaptic transmission and plasticity at adult synapses in many regions of the central nervous system. The NT actions are mediated by interacting with two transmembrane receptors with different affinity. Generally, all members of the NT family bind to pan-NT receptor p75NTR with low affinity, whereas mature NTs bind to different tropomyosin-related kinase (Trk) receptors, including TrkA, TrkB, and TrkC, with high affinity according to ligand selectivity. TrkA has been identified as the preferred receptor for NGF, and TrkB for BDNF [5]. The TrkB family comprisesseveral isoforms such as TrkB full-length (TrkB-FL), truncated TrkB.T1, and TrkB.T2. The intracellular region of TrkB has intrinsic tyrosine kinase activity. In contrast, TrkB.T1 lacks a tyrosine kinase domain and negatively regulated TrkB-FL [6]. BDNF plays essential roles in neuromuscular function. It was found that expression of BDNF, but not other members of the NT family, is perturbed in muscle from patients with Kennedy’s disease (KD). It was also reported that BDNF rescued synaptic and muscle function in a muscle-type specific manner in KD mice model [7].Here, we summarize the progresses of BDNF/TrkB-related signaling pathways and the critical roles in cardiovascular diseases (CVD) and discuss the potential therapeutic role of targeting BDNF/TrkB.

## 2. BDNF/TrkB-Related Signaling Pathways

To date, a variety of signaling pathways have been found to be regulated by BDNF/TrkB, such as PI3K/Akt, mitogen-activated protein kinase (MAPK), PLCγ, nuclear factor κB (NF-κB),and so forth [4]. Theoretically, activation of TrkBsimulates BDNF activity, including TrkB receptor phosphorylation and dimerization, as well asactivating Akt and MAPK pathways.For example, Gupta et al. reported that BDNF mimetic 7,8-dihydroxyflavone (7,8-DHF) activated the TrkB signaling and protected the retinal ganglion cells from excitotoxic and oxidative stress by activating the downstream cell survival Akt and extracellular-regulated protein kinase (ERK)1/2pathways [8]. Similarly, Kang et al. found that 7,8-DHF repressed hydrogen peroxide(H_2_O_2_)-induced damage of C2C12 myoblasts by scavenging reactive oxygen species (ROS) and activatingthe PI3K/Akt and ERK signaling pathways [9]. Huanget al. found that early systemic 7,8-DHF treatment protected immature retinas against hypoxic-ischemic injury by modulatingthe MAPK/ERK signaling pathway [10]. In contrast, it wasfound that ERK signals were not affected after 7,8-DHFtreatment [11], which contradicts previous studies. Moreover, other studies also reported inhibition of MAPK during the protection of 7,8-DHF. For instance, Park et al. found that 7,8-DHF inhibitedlipopolysaccharide-induced inflammation by negatively regulating NF-κB and MAPK activation [12].Likewise, another study supported that 7,8-DHF protected PC12 cells against the neurotoxin 6-OHDA-induced cytotoxicity via activating PI3K/Akt and inhibiting c-Jun N-terminal kinase (JNK) pathways [13].However, it should be noted that the majority of current findings are in the neurons, and mechanistic evidence regardingthe potential role of BDNF/TrkB in the cardiovascular system is limited. Our recent study indicated that 7,8-DHF activated the TrkB receptor and protected against cytotoxic anthracycline antibiotic doxorubicin (DOX)-induced cardiotoxicity by activating Akt and inhibiting ERK [14]. Moreover, we also demonstrated that 7,8-DHF attenuated ischemic myocardial injury by activating Akt and inhibited mitochondrial excessive fission [15]. More workshould be done to comprehensively elucidate the role of 7,8-DHF in the cardiovascular system, which would help us to better clarifythe molecular mechanisms of 7,8-DHF in both cardiac and vascular cells.

## 3. Role of BDNF/TrkB in Coronary Artery Diseases

**Figure 1:**
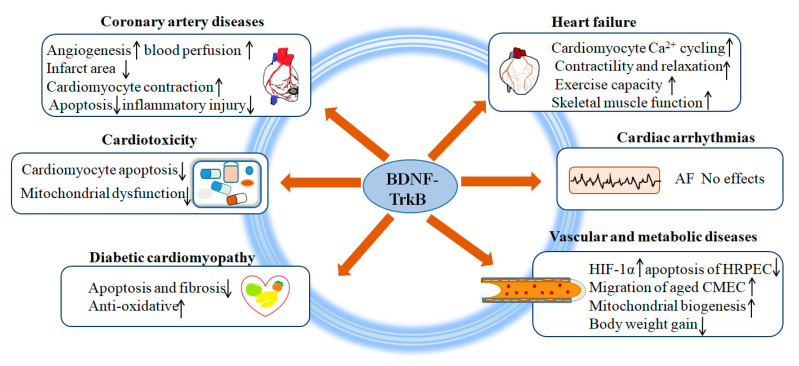
Schematic representation of BDNF/TrkB in cardiovascular diseases.AF, atrial fibrillation; HIF-1α, hypoxia inducible factor 1 alpha; HRPEC, human retinal pigment epithelial cells; CMEC, cardiac microvascular endothelial cells.

Because BDNF is a widely existing neurotrophic factor in brain tissue and peripheral nerves, it can affect nerve development, survival, and postinjury repair. Previous studies mainly focused on its role in neurological diseases and found that BDNF had protective effects against cerebral ischemic injury. Currently, more and more studies suggest that the BDNF/TrkB axis plays an important role in cardiovascular function. Here, we summarize current advancements of the critical role of BDNF/TrkB in common CVD (Figure 1).

Several previous studies firstly paid attention to theeffects of BDNF on myocardial infarction (MI). It was found that intramyocardial injection of BDNF post-MI induced angiogenesis in the infarct area and the peripheral area. The study also found that simultaneous injection of basic fibroblast growth factor (bFGF) and BDNF significantly increasedmicrovascular density in a canine infarct model and improved blood perfusion and cardiac function compared withbFGF appliedalone [16]. In fact, early studies mainly focused on the role of BDNF in neurological diseases and found that BDNF had protective effects on cerebral ischemic injury. For example, Schabitz et al. found that intracerebral injection of BDNF could effectively reduce the infarct area in ischemic rats [17]. Since BDNF and its receptor TrkB are also distributed in myocardial tissue, the role of BDNF in myocardial tissue has been gradually emphasized by researchers in recent years, and there is evidence that it has an obvious myocardial protective effect. Hiltunen et al. found that after myocardial ischemia–reperfusion for 2 and 5 h in rats, the expression of BDNF in the left ventricular myocardium increased by twofold, and the mRNA expression of BDNF could be detected in the ischemic marginal area [18]. Ejiri et al. found that BDNF significantly improved coronary heart disease, and the study found that the content of BDNF in plasma was significantly increased in patients with unstable angina pectoris [19]. Likewise, studiesby Okada et al.confirmed that the plasma level of BDNF in myocardial ischemicmice increased significantly, which puts forward the central nervous system-mediated signal transduction mechanism to protect ischemic myocardium: when there is cardiac ischemic damage, myocardial cells through efferent nerve feedback to the central nervous system increase the expression of BDNF in the nerve cells, so as to increase the level of BDNF in peripheral blood, activating theangiogenesis-related molecular mechanism of inhibiting myocardial reconstruction and repair of ischemic myocardium [20]. Recently, a new study also uncovered serum BDNF was differentially expressed in plaque tissues in patients with coronary heart disease compared with normal intimae in healthy subjects using microarray profiling analysis and qRT-PCR validation [21]. Recent studies have revealed that BDNF regulates cardiomyocyte contraction and directly affects cardiac function of mice [22,23]. Consistently, our previous studies found that exogenous BDNF attenuated myocardial ischemia by inhibiting apoptosis of rat cardiomyocytes [24]. However, other studies reported that BDNF may aggravate the inflammatory injury in the hearts of aging rats [25,26,27]. However, why BDNF plays different roles between young and old remains not fully understood. So, more work isneeded to further explore the role of BDNF/TrkB in cardiac inflammation and senescence. Based on these findings, the potential role of BDNF in ischemic myocardium is attracting more and more attention. However, the molecular mechanism of BDNF in protecting ischemic myocardium should be clarified by more solid evidence.

## 4. Role of BDNF/TrkB in Heart Failure (HF)

A study found that BDNF enhanced normal cardiomyocyte Ca^2+^ cycling, contractility, and relaxation via regulating Ca^2+^/calmodulin-dependent protein kinase II (CaMKII) signals. However, TrkB.T1 was increased in the failing rodent myocytes, which is insensitive to BDNF due to lack of tyrosine kinase activity [22]. Moreover, the majority of recent studiesfocused on the association of BDNF level inperipheral blood with progress and prognosis of HF (summarized below). Lately, studies found that BDNF was relevant to exercise in heart diseases. For example, Zhang et al. reported that exercise training amelioratedHF-induced muscle atrophy by activating BDNF and adenosine monophosphate-activated protein kinase-proliferator-activated receptor-γ coactivator-1α (AMPK-PGC1α) signaling pathway [28]. Exercise training after MI increased mature BDNF protein in both skeletal muscle and the noninfarct area of the left ventricles, and improved muscle dysfunction and cardiac function post-MI [29]. On the other hand, BDNF treatment also improved exercise capacity in HF mice [30]. A very recent study proved that the protective effects of BDNF on the exercise capacity of mice with HFwere due to the enhancement of fatty acid oxidation via the activation of the AMPKα-PGC1α pathway in skeletal muscle [31]. Serum BDNF levels were associated with exercise capacity and skeletal muscle function, but not with muscle mass in patients with HF. BDNF production was controlled by muscle function and activity and consequently regulated exercise capacity [32]. Exercise training post-MI increased mature BDNF in the paraventricular nucleus (PVN), but not in the rostral ventrolateral medulla (RVLM). Exercise decreased Akt activity in the PVN and p-CaMKIIβ expression in the RVLM, but BDNF-TrkB signaling only mediated the effect of exercise on p-CaMKIIβ in the RVLM, but not of Akt in the PVN [33].

## 5. Role of BDNF/TrkB in Cardiotoxicity

We previously found that BDNF/TrkB signaling was impaired in DOX-induced cardiotoxicity. Cardiac BDNF protein expression was lower in DOX-treated mice than control mice. Intravenous injection of recombinant human BDNF inhibited cardiac dysfunction and cardiomyocyte apoptosis by activating Akt signals [34]. Similar effectswere found in pancreatic β cells. It was observed that DOX significantly inhibited BDNF production with a dose-dependent manner, while BDNF prevented apoptosis and restored the antioxidant levels in rat insulinoma cells [35]. Furthermore, ourstudyfurther uncovered that BDNF mimetic, 7,8-DHF, activated the TrkB receptor and attenuated DOX-induced cardiotoxicity and mitochondrial dysfunction in a mouse model [14]. Consistently, Liao et al. reproduced that neurotrophic signaling including both BDNF/TrkB and NGF/TrkAwasaffected by DOX [36]. Besides, there is now consensus that a number of chemotherapy drugs such as arsenic trioxide cause cardiotoxicity. Arsenic trioxide has been confirmed to induce a long QT interval and cardiomyocyte apoptosis [37,38]. However, the effects of the BDNF/TrkB pathway in arsenic trioxide-induced cardiotoxicity wereunknown. So, it is worth studying the potential role of BDNF/TrkB in this drug-induced cardiotoxicity and further investigating the therapeutic implications.

## 6. Role of BDNF/TrkB in Diabetes

Studies have clarified that circulating BDNF level is decreased in type 2 diabetic patients. Replenishing BDNF or enhancing its downstream signaling pathway may be beneficial for diabetes [39]. Cardiac levels of BDNF and TrkB receptor were reduced in type 2 diabetic rats. *L. plantarum* and insulin supplementation inhibited cardiac apoptosis and fibrosis by modulating BDNF and enhancing TrkB expression [40]. In addition, caveolin-3 overexpression was reported to protect diabetic rat cardiomyocytes against acute MI/reperfusion injury by enhancing BDNF/TrkB signaling pathways [41]. It was documented that NTs play important roles in the regulation of energy balance and body weight [42,43]. In particular, BDNF treatment enhanced energy expenditure in db/dbmice by acting directly on the hypothalamus [44]. BDNF ameliorated glucose metabolism by enhancement of glucose utilization in muscle and brown adipose tissue of diabetic mice, via modulating the central and peripheral nervous systems [45].

## 7. Role of BDNF/TrkB in Cardiac Arrhythmias

Studies have demonstrated that BDNF/TrkB signaling is required for normal cardiac contraction and relaxation. Fulgenzi et al. reported thatcardiac BDNF maintained heart contraction force and long-term homeostasis through TrkB.T1. Deletion of TrkB.T1 in the cardiomyocytes caused dysregulated calcium signaling and cardiomyopathy [23]. In contrast, few studies reported a relationship between BDNF and cardiac arrhythmias. In a prospective study, Rahman et al. explored whether BDNF was associated with incidence of atrial fibrillation (AF). They found that serum BDNF level was not statistically related with AF occurrence [46]. However, the roles of BDNF in ventricular arrhythmias are still largely unknown.

## 8. Role of BDNF/TrkB in Vascular and Metabolic Diseases

It wasreported that intravenous treatment of 7,8-DHF produced an antihypertensive effect in spontaneously hypertensive rats due to its vasodilation role [47]. Another study by Helan et al. reported that human pulmonary artery endothelial cells expressed and secreted BDNF in response to hypoxia, which induced expression of hypoxia inducible factor 1 alpha (HIF-1α) [48]. Wood et al. demonstrated that BDNF mimetic 7,8-DHF treatment increased cellular respiration of skeletal muscle cells by facilitating mitochondrial biogenesis, thereby reduced body weight gain in obese mice [49]. Consistently, Chan et al. found that activation of the muscular TrkB receptor regulated energy metabolism in diet-induced obese mice [50]. Moreover, other studies also found that activation of the TrkB receptor alleviated complication of diabetes. For instance, Allen et al. reported that the TrkB signaling pathway is indispensable in the protective effects of exercise against the diabetic retina complication [51]. Agrimi et al. reported cardiac dysfunction characterized by apoptosis, fibrosis, and oxidative stress in obese mice exposed to psychosocial stress. Simultaneously, lower BDNF/TrkB level was observed in mouse ventricles [52]. However, the authors did not provide direct causal evidence supporting BDNF/TrkB contributed to cardiac remodeling. Lately, another study found that 7,8-DHF protected neurons from high glucose-induced oxidative stress [53]. However, the potential role of 7,8-DHF in high glucose-induced cardiomyocyte injury and diabetic cardiomyopathy is undetermined. Yu et al. reported that activation of the TrkB receptor by 7,8-DHF repressed apoptosis of human retinal pigment epithelial cells [54]. Recent studies also found that the truncated TrkB-T1 is the unique isoform expressed in aged cardiac microvascular endothelial cells (CMECs). Meanwhile, BDNF activated TrkB.T1 and thereby induced migration of aged CMECs by recruiting the Willin–Hippo pathway [55].

## 9. Role of BDNF/TrkB in Noncardiac Cells

It should be noted that BDNF and TrkB are expressed in various cell types such as cardiomyocytes, endothelial cells (ECs), vascular smooth muscle cells (VSMCs), and fibroblasts in the cardiovascular system, thus the potential role of BDNF/TrkB in noncardiac cells should also be seriously considered. In particular, it was noticed that BDNF/TrkB participated in the activities of fibroblasts. For example, Cherubini and colleagues found that TrkB was expressed in lung fibroblasts, and activation of BDNF/TrkB axis promoted the epithelial–mesenchymal transition in idiopathic pulmonary fibrosis [56]. Similarly, BDNF/TrkB was associated with pathologic changes of other types of fibroblasts such as airway [57] and human dermal fibroblast cells [58]. Accordingly, whether BDNF/TrkB is involved in cardiac interstitial fibrosis is interesting to further investigate. In addition, BDNF is also expressed in pulmonary artery endothelial cells (PAECs) and gastric smooth muscle cells. Helan et al. reported that PAECs expressed and secreted BDNF in response to hypoxia via an HIF-1-regulated pathway [48]. Activation of the BDNF/TrkB axis accelerated epithelial–mesenchymal transition in idiopathic pulmonary fibrosis [56]. In addition, recent studies found that 7,8-DHF enhanced cholinergic contraction of rat gastric smooth muscle via upregulating muscarinic M_3_ receptor expression [59]. The detailed role of BDNF/TrkB in these cells will be uncovered in the future.

## 10. Role of Circulating BDNF in CVD

Several clinical studies paid attention to the association of circulating BDNF in either plasma or serum with CVD, including coronary artery disease, angina, HF, and diabetes. In 2005, Ejiri et al. firstly reported that coronary sinus and aorta BDNF level was significantly higher in the unstable angina group than the stable effort angina and noncoronary artery disease groups [19]. It was reported that serum BDNF was significantly lower in individuals with CAD than controls [60]. Higher serum BDNF is associated with a decreased risk of CVD and mortality [61]. Moreover, serum BDNF was found correlated with HF. For example, Takashio et al. found that plasma BDNF levels were decreased in patients with HF and associated with HF severity [62]. Studies demonstrated that decreased serum BDNF levels were significantly associated with adverse outcomes in HF patients [63,64]. Recent studies also found that the serum BDNF level at discharge may be a useful biomarker of the prognosis in patients with HF [65]. They proposed that serum BDNF can be a useful prognostic biomarker to predict adverse clinical outcomes in patients with HF.Besides, decreased serum BDNF levels are correlated with exercise intolerance in patients with HF [66]. Further studies found that BDNF levels were associated with exercise capacity and skeletal muscle function, but not with muscle mass [32]. In contrast, no significant association of serum BDNF levels and the risk of incident AFwas found [46]. In addition, plasma BDNF was decreased in aged rats compared to young rats, which could be increased by regular exercise [67]. Interestingly, a recent study reported that serum BDNF, as well as irisin andadropin, is correlated with the occurrence of depression in coronary heart disease patients [68]. A recent study also found that circulatory BDNF was decreased in patients with type 2 diabetes and depression [69]. Taken together, circulating BDNF may be a candidate biomarker for some cardiac diseases (summarized in Table 1). More studies are needed to compare the sensitivity, specificity, accuracy, and correlation with currently used markers to better evaluate its feasibility in clinical diagnosis and treatment.

## 11. Concluding Remarks

At present, studies on BDNF structure, molecular morphology, action, and mechanism have achieved stage achievements. The emerging role of BDNF/TrkB in CVD was paid more and more attention. Several clinical applications of BDNF have been developed, which can treat common neuropsychiatric diseases, such as Parkinson’s disease, Alzheimer’s disease, depression, schizophrenia, and so forth [70,71,72,73], and can also be used as a reference indicator for early diagnosis and prognosis of certain tumors [74]. However, BDNF itself has the following limitations:(1) short half-life, low tissue content, rapid metabolism after entering the body, and difficulty in reaching sufficient drug concentration and maintenance time; (2) production technology requirements and the high cost and low yield limit its use as a clinical conventional drug. However, gene engineering technology can transfer the gene fragment expressing BDNF to the expression cells through vectors, so as to achieve the effect of local sustained release of BDNF and avoid a lot of inconvenience such as drug administration. Recent studies have also linked a segment of highly charged, alkaline amino acid transactivator of transcription (TAT) encoded by human immunodeficiency virus type 1 (HIV-1) to the BDNF to improve its transmission through the blood–brain barrier, and the results show that TAT-BDNF can effectively target the brain and improve cognitive function in animals with Alzheimer’s disease [75]. In addition, several BDNF mimics/TrkB agonists (e.g., 7,8-DHF) have been discovered to simulate BDNF action. Meanwhile, numerous derivatives of TrkB agonists have been developed to examine their efficacy [76]. With the deepening of research and the continuous development of new technologies, the technical bottleneck in the BDNF translation is expected to be overcome [77,78].

## Figures and Tables

**Table 1:** Circulating BDNF as a potential biomarker in cardiovascular diseases.

Diseases	Source	Description	References
Unstable angina	Plasma	Higher in unstable angina compared with the stable effort angina and noncoronary artery disease groups	Ejiri J et al. 2005 [19]
Coronary artery disease	Serum	BDNF was significantly lower in patients with coronary artery disease than control	Monisha K et al. 2020 [60]
Myocardial ischemia	Plasma	BDNF was markedly increased after myocardial ischemia	Okada S et al. 2012 [20]
Cardiovascular diseases	Serum	Higher BDNF level is associated with a decreased risk of cardiovascular diseases and mortality	Kaess B et al. 2015 [61]
Depression in coronary heart disease	Plasma	Plasma BDNF was associated with occurrence of depression in coronary heart disease	Han W et al. 2019 [68]
Heart failure	Plasma	Low BDNF level is associated with the severity of heart failure	Takashio S et al. 2015 [62]
Heart failure	Serum	BDNF predicts adverse clinical outcomes in patients with heart failure	Fukushima A et al. 2015 [63]
Heart failure	Serum	Low BDNF level is independently associated with an increased risk of cardiac events	Kadowaki S et al. 2015 [64]
Heart failure	Serum	BDNF level at discharge may be a useful biomarker of the prognosis in patients with heart failure	Shibata A et al. 2018 [65]
Heart failure	Serum	Decreased BDNF is correlated with exercise intolerance in patients with heart failure	Fukushima A et al. 2013 [66]
Heart failure	Serum	BDNF levels were associated with exercise capacity and skeletal muscle function, but not with muscle mass	Nakano I et al. 2020 [32]
Atrial fibrillation	Serum	No statistically significant association was found between serum BDNF levels and risk of incident atrial fibrillation	Rahman F et al. 2016 [46]
Aging	Plasma	Regular exercise improves aging-induced decrease in the cardiac, hepatic, and plasma BDNF	Belviranli M et al. 2018 [67]
T2DM with depression	Serum	Decreased levels of circulatory BDNF in patients with type 2 diabetes and depression	Prabu P et al. 2020 [69]

## Abbreviations

BDNF	brain-derived neurotrophic factor
CVD	cardiovascular diseases
DOX	doxorubicin
7,8-DHF	7,8-dihydroxyflavone
ERK	extracellular regulated protein kinase
HF	heart failure
MAPK	mitogen-activated protein kinase
MI	myocardial infarction
NF-κB	nuclear factor κB
NGF	neuronal growth factor
NT	neurotrophin
TrkB	tropomyosin-related kinase receptor B

## References

[1] Chao M.V., Rajagopal R., Lee F.S. (2006). Neurotrophin signalling in health and disease. Clin. Sci..

[2] Barde Y.A., Edgar D., Thoenen H. (1982). Purification of a new neurotrophic factor from mammalian brain. EMBO J..

[3] Scarisbrick I.A., Jones E.G., Isackson P.J. (1993). Coexpression of mRNAs for NGF, BDNF, and NT-3 in the cardiovascular system of the pre- and postnatal rat. J. Neurosci..

[4] Numakawa T., Suzuki S., Kumamaru E., Adachi N., Richards M., Kunugi H. (2010). BDNF function and intracellular signaling in neurons. Histol. Histopathol..

[5] László A., Lénárt L., Illésy L., Fekete A., Nemcsik J. (2019). The role of neurotrophins in psychopathology and cardiovascular diseases: Psychosomatic connections. J. Neural Transm..

[6] Gupta V., You Y., Gupta V.B., Klistorner A., Graham S.L. (2013). TrkB Receptor Signalling: Implications in Neurodegenerative, Psychiatric and Proliferative Disorders. Int. J. Mol. Sci..

[7] Halievski K., Xu Y., Haddad Y.W., Tang Y.P., Yamada S., Katsuno M., Adachi H., Sobue G., Breedlove S.M., Jordan C.L. (2020). Muscle BDNF improves synaptic and contractile muscle strength in Kennedy’s disease mice in a muscle-type specific manner. J. Physiol..

[8] Gupta V.K., You Y., Li J.C., Klistorner A., Graham S.L. (2013). Protective Effects of 7,8-Dihydroxyflavone on Retinal Ganglion and RGC-5 Cells Against Excitotoxic and Oxidative Stress. J. Mol. Neurosci..

[9] Kang J.S., Choi I.-W., Han M.H., Kim G.-Y., Hong S.H., Park C., Hwang H.J., Kim C.M., Kim B.W., Choi Y.H. (2015). The cytoprotective effects of 7,8-dihydroxyflavone against oxidative stress are mediated by the upregulation of Nrf2-dependent HO-1 expression through the activation of the PI3K/Akt and ERK pathways in C2C12 myoblasts. Int. J. Mol. Med..

[10] Huang H.-M., Huang C.-C., Tsai M.-H., Poon L.Y.-C., Chang Y.-C. (2018). Systemic 7,8-Dihydroxyflavone Treatment Protects Immature Retinas Against Hypoxic-Ischemic Injury via Müller Glia Regeneration and MAPK/ERK Activation. Investig. Opthalmol. Vis. Sci..

[11] Tsai T., Klausmeyer A., Conrad R., Gottschling C., Leo M., Faissner A., Wiese S. (2013). 7,8-Dihydroxyflavone leads to survival of cultured embryonic motoneurons by activating intracellular signaling pathways. Mol. Cell. Neurosci..

[12] Park H.Y., Kim G.Y., Hyun J.W., Hwang H.J., Kim N.D., Kim B.W., Choi Y.H. (2012). 7,8-Dihydroxyflavone exhibits anti-inflammatory properties by downregulating the NF-kappaB and MAPK signaling pathways in lipopolysaccharide-treated RAW264.7 cells. Int. J. Mol. Med..

[13] Han X., Cheng M.-N., Chen L., Fang H., Wang L.-J., Li X.-T., Qu Z.-Q. (2014). 7,8-Dihydroxyflavone protects PC12 cells against 6-hydroxydopamine-induced cell death through modulating PI3K/Akt and JNK pathways. Neurosci. Lett..

[14] Zhao J., Du J., Pan Y., Chen T., Zhao L., Zhu Y., Chen Y., Zheng Y., Liu Y., Sun L. (2019). Activation of cardiac TrkB receptor by its small molecular agonist 7,8-dihydroxyflavone inhibits doxorubicin-induced cardiotoxicity via enhancing mitochondrial oxidative phosphorylation. Free Radic. Biol. Med..

[15] Wang Z., Wang S.-P., Shao Q., Li P.-F., Sun Y., Luo L.-Z., Yan X.-Q., Fan Z.-Y., Hu J., Zhao J. (2019). Brain-derived neurotrophic factor mimetic, 7,8-dihydroxyflavone, protects against myocardial ischemia by rebalancing optic atrophy 1 processing. Free Radic. Biol. Med..

[16] Liu Y., Sun L., Huan Y., Zhao H., Deng J. (2006). Application of bFGF and BDNF to Improve Angiogenesis and Cardiac Function. J. Surg. Res..

[17] Schäbitz W.R., Steigleder T., Cooper-Kuhn C.M., Schwab S., Sommer C., Schneider A., Kuhn H.G. (2007). Intravenous Brain-Derived Neurotrophic Factor Enhances Poststroke Sensorimotor Recovery and Stimulates Neurogenesis. Stroke.

[18] Hiltunen J.O., Laurikainen A., Meri S., Saarma M. (2001). Nerve growth factor and brain-derived neurotrophic factor mRNAs are regulated in distinct cell populations of rat heart after ischaemia and reperfusion. J. Pathol..

[19] Ejiri J., Inoue N., Kobayashi S., Shiraki R., Otsui K., Honjo T., Takahashi M., Ohashi Y., Ichikawa S., Terashima M. (2005). Possible Role of Brain-Derived Neurotrophic Factor in the Pathogenesis of Coronary Artery Disease. Circulation.

[20] Okada S., Yokoyama M., Toko H., Tateno K., Moriya J., Shimizu I., Nojima A., Ito T., Yoshida Y., Kobayashi Y. (2012). Brain-Derived Neurotrophic Factor Protects Against Cardiac Dysfunction After Myocardial Infarction via a Central Nervous System–Mediated Pathway. Arter. Thromb. Vasc. Biol..

[21] Bai H.-L., Lu Z.-F., Zhao J.-J., Ma X., Li X.-H., Xu H., Wu S.-G., Kang C.-M., Lu J.-B., Xu Y.-J. (2019). Microarray profiling analysis and validation of novel long noncoding RNAs and mRNAs as potential biomarkers and their functions in atherosclerosis. Physiol. Genom..

[22] Feng N., Huke S., Zhu G., Tocchetti C.G., Shi S., Aiba T., Kaludercic N., Hoover D.B., Beck S.E., Mankowski J.L. (2015). Constitutive BDNF/TrkB signaling is required for normal cardiac contraction and relaxation. Proc. Natl. Acad. Sci. USA.

[23] Fulgenzi G., Tomassoni-Ardori F., Babini L., Becker J., Barrick C., Puverel S., Tessarollo L. (2015). BDNF modulates heart contraction force and long-term homeostasis through truncated TrkB.T1 receptor activation. J. Cell Biol..

[24] Hang P., Zhao J., Cai B., Tian S., Huang W., Guo J., Sun C., Li Y., Du Z. (2015). Brain-derived neurotrophic factor regulates TRPC3/6 channels and protects against myocardial infarction in rodents. Int. J. Biol. Sci..

[25] Cao L., Zhang L., Chen S., Yuan Z., Liu S., Shen X., Zheng X., Qi X., Lee K.K., Chan J.Y.-H. (2012). BDNF-mediated migration of cardiac microvascular endothelial cells is impaired during ageing. J. Cell. Mol. Med..

[26] Halade G.V., Ma Y., Ramirez T.A., Zhang J., Dai Q., Hensler J.G., Lopez E.F., Ghasemi O., Jin Y.-F., Lindsey M.L. (2013). Reduced BDNF attenuates inflammation and angiogenesis to improve survival and cardiac function following myocardial infarction in mice. Am. J. Physiol. Circ. Physiol..

[27] Cai D., Holm J.M., Duignan I.J., Zheng J., Xaymardan M., Chin A., Ballard V.L.T., Bella J.N., Edelberg J.M. (2006). BDNF-mediated enhancement of inflammation and injury in the aging heart. Physiol. Genom..

[28] Zhang Z., Wang B., Fei A. (2018). BDNF contributes to the skeletal muscle anti-atrophic effect of exercise training through AMPK-PGC1α signaling in heart failure mice. Arch. Med. Sci..

[29] Lee H.W., Ahmad M., Wang H.-W., Leenen F.H.H. (2017). Effects of exercise training on brain-derived neurotrophic factor in skeletal muscle and heart of rats post myocardial infarction. Exp. Physiol..

[30] Matsumoto J., Takada S., Kinugawa S., Furihata T., Nambu H., Kakutani N., Tsuda M., Fukushima A., Yokota T., Tanaka S. (2018). Brain-Derived Neurotrophic Factor Improves Limited Exercise Capacity in Mice With Heart Failure. Circulation.

[31] Matsumoto J., Takada S., Furihata T., Nambu H., Kakutani N., Maekawa S., Mizushima W., Nakano I., Fukushima A., Yokota T. (2020). Brain-Derived Neurotrophic Factor Improves Impaired Fatty Acid Oxidation Via the Activation of Adenosine Monophosphate-activated Protein Kinase-α—Proliferator-Activated Receptor-r Coactivator-1α Signaling in Skeletal Muscle of Mice With Heart Failure. Circ. Heart Fail..

[32] Nakano I., Kinugawa S., Hori H., Fukushima A., Yokota T., Takada S., Kakutani N., Obata Y., Yamanashi K., Anzai T. (2020). Serum Brain-Derived Neurotrophic Factor Levels Are Associated with Skeletal Muscle Function but Not with Muscle Mass in Patients with Heart Failure. Int. Heart J..

[33] Lee H.W., Ahmad M., Wang H.W., Leenen F.H.H. (2020). Effects of exercise on BDNF-TrkB signaling in the paraventricular nucleus and rostral ventrolateral medulla in rats post myocardial infarction. Neuropeptides.

[34] Hang P., Zhao J., Sun L., Li M., Han Y., Du Z., Li Y. (2016). Brain-derived neurotrophic factor attenuates doxorubicin-induced cardiac dysfunction through activating Akt signalling in rats. J. Cell. Mol. Med..

[35] Bathina S., Srinivas N., Das U.N. (2016). BDNF protects pancreatic beta cells (RIN5F) against cytotoxic action of alloxan, streptozotocin, doxorubicin and benzo(a)pyrene in vitro. Metab. Clin. Exp..

[36] Liao D., Zhang C., Liu N., Cao L., Wang C., Feng Q., Yao D., Long M., Jiang P. (2019). Involvement of neurotrophic signaling in doxorubicin‑induced cardiotoxicity. Exp. Ther. Med..

[37] Zhao X.-Y., Li G.-Y., Liu Y., Chai L.-M., Chen J.-X., Zhang Y., Du Z.-M., Lu Y.-J., Yang B. (2008). Resveratrol protects against arsenic trioxide-induced cardiotoxicity in vitro and in vivo. Br. J. Pharmacol..

[38] Zhao X., Feng T., Chen H., Shan H., Zhang Y., Lu Y., Yang B. (2008). Arsenic Trioxide-Induced Apoptosis in H9c2 Cardiomyocytes: Implications in Cardiotoxicity. Basic Clin. Pharmacol. Toxicol..

[39] Chan C.B., Ahuja P., Ye K. (2019). Developing Insulin and BDNF Mimetics for Diabetes Therapy. Curr. Top. Med. Chem..

[40] Sefidgari-Abrasi S., Roshangar L., Karimi P., Morshedi M., Rahimiyan-Heravan M., Saghafi-Asl M. (2021). From the gut to the heart: L. plantarum and inulin administration as a novel approach to control cardiac apoptosis via 5-HT2B and TrkB receptors in diabetes. Clin. Nutr..

[41] Gong J., Zhou F., Wang S.X.X., Xu J., Xiao F. (2020). Caveolin-3 protects diabetic hearts from acute myocardial infarction/reperfusion injury through beta2AR, cAMP/PKA, and BDNF/TrkB signaling pathways. Aging.

[42] Xu B., Xie X. (2016). Neurotrophic factor control of satiety and body weight. Nat. Rev. Neurosci..

[43] Rios M. (2014). Neurotrophins and the Regulation of Energy Balance and Body Weight. Lipid Signal. Hum. Dis..

[44] Nakagawa T., Ono-Kishino M., Sugaru E., Yamanaka M., Taiji M., Noguchi H. (2002). Brain-derived neurotrophic factor (BDNF) regulates glucose and energy metabolism in diabetic mice. Diabetes/Metab. Res. Rev..

[45] Yamanaka M., Tsuchida A., Nakagawa T., Nonomura T., Ono-Kishino M., Sugaru E., Noguchi H., Taiji M. (2007). Brain-derived neurotrophic factor enhances glucose utilization in peripheral tissues of diabetic mice. Diabetes Obes. Metab..

[46] Rahman F., Himali J.J., Yin X., Beiser A.S., Ellinor P.T., Lubitz S.A., Vasan R.S., Magnani J.W., McManus D.D., Seshadri S. (2016). Serum brain-derived neurotrophic factor and risk of atrial fibrillation. Am. Heart J..

[47] Huai R., Han X., Wang B., Li C., Niu Y., Li R., Qu Z. (2013). Vasorelaxing and Antihypertensive Effects of 7,8-Dihydroxyflavone. Am. J. Hypertens..

[48] Helan M., Aravamudan B., Hartman W.R., Thompson M.A., Johnson B.D., Pabelick C.M., Prakash Y.S. (2014). BDNF secretion by human pulmonary artery endothelial cells in response to hypoxia. J. Mol. Cell. Cardiol..

[49] Wood J., Tse M.C.L., Yang X., Brobst D., Liu Z., Pang B.P.S., Chan W.S., Zaw A.M., Chow B.K., Ye K. (2018). BDNF mimetic alleviates body weight gain in obese mice by enhancing mitochondrial biogenesis in skeletal muscle. Metab. Clin. Exp..

[50] Chan C.B., Tse M.C.L., Liu X., Zhang S., Schmidt R., Otten R., Liu L., Ye K. (2015). Activation of Muscular TrkB by its Small Molecular Agonist 7,8-Dihydroxyflavone Sex-Dependently Regulates Energy Metabolism in Diet-Induced Obese Mice. Chem. Biol..

[51] Allen R.S., Hanif A.M., Gogniat M.A., Prall B.C., Haider R., Aung M.H., Prunty M.C., Mees L.M., Coulter M.M., Motz C.T. (2018). TrkB signalling pathway mediates the protective effects of exercise in the diabetic rat retina. Eur. J. Neurosci..

[52] Agrimi J., Spalletti C., Baroni C., Keceli G., Zhu G., Caragnano A., Matteucci M., Chelko S.P., Ramirez-Correa G.A., Bedja D. (2019). Obese mice exposed to psychosocial stress display cardiac and hippocampal dysfunction associated with local brain-derived neurotrophic factor depletion. EBioMedicine.

[53] Cho S.J., Kang K.A., Piao M.J., Ryu Y.S., Fernando P.D.S.M., Zhen A.X., Hyun Y.J., Ahn M.J., Kang H.K., Hyun J.W. (2019). 7,8-Dihydroxyflavone Protects High Glucose-Damaged Neuronal Cells against Oxidative Stress. Biomol. Ther..

[54] Yu X., Liu Q., Wang X., Liu H., Wang Y. (2018). 7,8-Dihydroxyflavone ameliorates high-glucose induced diabetic apoptosis in human retinal pigment epithelial cells by activating TrkB. Biochem. Biophys. Res. Commun..

[55] Wang Z., Chen Y., Chen X., Zheng X., Xu G., Yuan Z., Zhao H., Chen W., Li L., Zheng N. (2019). The TrkB-T1 receptor mediates BDNF-induced migration of aged cardiac microvascular endothelial cells by recruiting Willin. Aging Cell.

[56] Cherubini E., Mariotta S., Scozzi D., Mancini R., Osman G., D’Ascanio M., Bruno P., Cardillo G., Ricci A. (2017). BDNF/TrkB axis activation promotes epithelial–mesenchymal transition in idiopathic pulmonary fibrosis. J. Transl. Med..

[57] Freeman M.R., Sathish V., Manlove L., Wang S., Britt R.D., Thompson M.A., Pabelick C.M., Prakash Y.S. (2017). Brain-derived neurotrophic factor and airway fibrosis in asthma. Am. J. Physiol. Cell. Mol. Physiol..

[58] Choi J.W., Lee J., Park Y.I. (2017). 7,8-Dihydroxyflavone attenuates TNF-α-induced skin aging in Hs68 human dermal fibroblast cells via down-regulation of the MAPKs/Akt signaling pathways. Biomed. Pharmacother..

[59] He B., Qu Z., Tian Z., Zhao K., Wei L., Ma L. (2018). 7,8-dihydroxyflavone enhanced cholinergic contraction of rat gastric smooth muscle via augmenting muscarinic M3 receptor expression. Clin. Exp. Pharmacol. Physiol..

[60] Monisha K.G., Prabu P., Chokkalingam M., Murugesan R., Milenkovic D., Ahmed S.S. (2020). Clinical utility of brain-derived neurotrophic factor as a biomarker with left ventricular echocardiographic indices for potential diagnosis of coronary artery disease. Sci. Rep..

[61] Kaess B.M., Preis S.R., Lieb W., Beiser A.S., Yang Q., Chen T.C., Hengstenberg C., Erdmann J., Schunkert H., Seshadri S. (2015). Circulating brain-derived neurotrophic factor concentrations and the risk of cardiovascular disease in the community. J. Am. Heart Assoc..

[62] Takashio S., Sugiyama S., Yamamuro M., Takahama H., Hayashi T., Sugano Y., Izumiya Y., Hokimoto S., Minamino N., Yasuda S. (2015). Significance of Low Plasma Levels of Brain-Derived Neurotrophic Factor in Patients With Heart Failure. Am. J. Cardiol..

[63] Fukushima A., Kinugawa S., Homma T., Masaki Y., Furihata T., Yokota T., Matsushima S., Takada S., Kadoguchi T., Oba K. (2015). Serum Brain-Derived Neurotropic Factor Level Predicts Adverse Clinical Outcomes in Patients With Heart Failure. J. Card. Fail..

[64] Kadowaki S., Shishido T., Honda Y., Narumi T., Otaki Y., Kinoshita D., Nishiyama S., Takahashi H., Arimoto T., Miyamoto T. (2015). Additive clinical value of serum brain-derived neurotrophic factor for prediction of chronic heart failure outcome. Heart Vessel..

[65] Shibata A., Hanatani A., Izumi Y., Kitada R., Iwata S., Yoshiyama M. (2018). Serum brain-derived neurotrophic factor level and exercise tolerance complement each other in predicting the prognosis of patients with heart failure. Heart Vessel..

[66] Fukushima A., Kinugawa S., Homma T., Masaki Y., Furihata T., Yokota T., Matsushima S., Abe T., Suga T., Takada S. (2013). Decreased serum brain-derived neurotrophic factor levels are correlated with exercise intolerance in patients with heart failure. Int. J. Cardiol..

[67] Belviranlı M., Okudan N. (2018). Exercise training increases cardiac, hepatic and circulating levels of brain-derived neurotrophic factor and irisin in young and aged rats. Horm. Mol. Biol. Clin. Investig..

[68] Han W., Zhang C., Wang H., Yang M., Guo Y., Li G., Zhang H., Wang C., Chen D., Geng C. (2019). Alterations of irisin, adropin, preptin and BDNF concentrations in coronary heart disease patients comorbid with depression. Ann. Transl. Med..

[69] Prabu P., Poongothai S., Shanthirani C.S., Anjana R.M., Mohan V., Balasubramanyam M. (2020). Altered circulatory levels of miR-128, BDNF, cortisol and shortened telomeres in patients with type 2 diabetes and depression. Acta Diabetol..

[70] Pollock K., Dahlenburg H., Nelson H., Fink K.D., Cary W., Hendrix K., Annett G., Torrest A., Deng P., Gutierrez J. (2016). Human Mesenchymal Stem Cells Genetically Engineered to Overexpress Brain-derived Neurotrophic Factor Improve Outcomes in Huntington’s Disease Mouse Models. Mol. Ther. J. Am. Soc. Gene Ther..

[71] Li Y., Xu J., Xu P., Song S., Liu P., Chi T., Ji X., Jin G., Qiu S., Hou Y. (2016). Xanthoceras sorbifolia extracts ameliorate dendritic spine deficiency and cognitive decline via upregulation of BDNF expression in a rat model of Alzheimer’s disease. Neurosci. Lett..

[72] Jiang B., Wang Y.-J., Wang H., Song L., Huang C., Zhu Q., Wu F., Zhang W. (2016). Antidepressant-like effects of fenofibrate in mice via the hippocampal brain-derived neurotrophic factor signalling pathway. Br. J. Pharmacol..

[73] Zhang X.Y., da Chen C., Tan Y.L., Tan S., Luo X., Zuo L., Soares J.C. (2016). BDNF Polymorphisms Are Associated With Cognitive Performance in Schizophrenia Patients Versus Healthy Controls. J. Clin. Psychiatry.

[74] Wu Y., Si R., Yang S., Xia S., He Z., Wang L., He Z., Wang Q., Tang H. (2016). Depression induces poor prognosis associates with the down-regulation brain derived neurotrophic factor of serum in advanced small cell lung cancer. Oncotarget.

[75] Wu Y., Luo X., Liu X., Liu D., Wang X., Guo Z., Zhu L., Tian Q., Yang X., Wang J.-Z. (2015). Intraperitoneal Administration of a Novel TAT-BDNF Peptide Ameliorates Cognitive Impairments via Modulating Multiple Pathways in Two Alzheimer’s Rodent Models. Sci. Rep..

[76] Chen C., Wang Z., Zhang Z., Liu X., Kang S.S., Zhang Y., Ye K. (2018). The prodrug of 7,8-dihydroxyflavone development and therapeutic efficacy for treating Alzheimer’s disease. Proc. Natl. Acad. Sci. USA.

[77] Bathina S., Das U.N. (2015). Brain-derived neurotrophic factor and its clinical implications. Arch. Med. Sci..

[78] Sanada K., Zorrilla I., Iwata Y., Bermúdez-Ampudia C., Graff-Guerrero A., Martínez-Cengotitabengoa M., González-Pinto A. (2016). The Efficacy of Non-Pharmacological Interventions on Brain-Derived Neurotrophic Factor in Schizophrenia: A Systematic Review and Meta-Analysis. Int. J. Mol. Sci..

